# Treatment of Acute Rheumatism

**Published:** 1865-06

**Authors:** 


### TREATMENT OF ACUTE RHEUMATISM.

Dr. Robert Law, Prof. Inst. Med. and Clin. Med. in School of Physic in Ireland, describes (*Dublin Quar. Jour. of Med. Sciences*, May, 1864) his mode of treatment of acute rheumatism, which he asserts to be more successful than any other. It consists in a moderate venesection, almost never exceeding eight ounces, and seldom requiring to be repeated; and in the exhibition of colchicum, either in the form of the tincture or the wine of the seeds, of which preparation he does not exceed a drachm in a six-drachm mixture, or the acetous extract in grain doses; three or four times daily. "When I

consider it necessary to exhibit an aperient, which I avoid as much as possible in such cases, from the pain of the motion consequent upon the operation of the medicine, I direct the following mixture: Tincture of the seeds of colchicum, one drachm; tincture of senna, half an ounce; sulphate of magnesia, six drachms; peppermint water to six ounces. I have found considerable advantage and ease to the patient from combining opium largely with the colchicum. I have already alluded to the fact of how very unsusceptible to the influence of opium persons affected with acute rheumatism are; it is quite remarkable what an amount of it they will bear without being narcotized. I constantly direct a drachm of the tincture of the seeds of colchicum, and a drachm of liquor opii sedative, in a six-ounce mixture, or a grain of the acetous extract of colchicum, and a grain of the watery extract of opium in a pill, three times or oftener in the day.

"Thus have I combined Dr. Corrigan's narcotic treatment of the disease with my own. The local application to the inflamed joints which I have employed with the most advantage is the tincture of iodine, and especially where there is effusion into the joints, which, in most cases, disappears speedily under its use. I have generally observed, where the pericardium or endocardium is about to be affected there is, in general, previously an excited action of the organ, in which case I add digitalis either to the mixture or pill. And when an attrition murmur, or a valvular abnormal sound, indicates pericarditis, or endocarditis, I then combine mercury with the other medicines in the following formula: Acetous extract of colchicum, four grains; calomel, three grains; watery extract of opium, two grains; powdered digitalis, one grain. To be made into four pills; one to be taken every third hour. I also direct a blister to be applied to the precordial region, and the blistered surface to be dressed with mercurial ointment, in order to bring the system speedily under the influence of this medicine—convinced as I am of its power to effect the absorption of the effused lymph, whether deposited on the pericardium, or on the surface or in the substance, whether superficially or interstitially, in the valves. This is the stage of the disease when medicine can alone cure it.

If, however, as is often the case, the valvular murmur be overlooked, and the heart not be suspected of being involved in the general rheumatic affection, and consequently the suitable treatment not have been directed against the complication the lymph allowed to run its unhindered pathological course, and in the exercise of its contractile property, permanently damages the valvular apparatus, the consequences of which injury become the objects of future treatment. Many question that it is in the power of medicine to restore the integrity of a valve which has been once diseased, and would rather believe that an abnormal sound which they supposed to indicate organic disease, now from the fact of its ceasing, was only functional or independent of structural change. As long as I am satisfied that mercury will promote the absorption of the lymph deposited in iritis; as long as I believe that a hepatized lung will return to its original condition as soon as the system is brought under the influence of mercury, so long will I cherish the conviction that mercury will do as much for the valves of the heart, which, if not exactly identical in structure with those parts I have alluded to, are at least analogous both in structure, pathological deportment, and therapeutic susceptibilities. And, under this conviction, we would explain the ceasing of the abnormal sound by the mercury having removed that which caused it. I have thought it necessary to dwell on this point of cardiac therapeutics, as I deem it of the utmost practical importance. I would also remark on the employment of digitalis in this early stage of pericarditis and of valvular disease. I have already observed how pericarditis is often preceded by an excited action of the organ. At this stage of the disease I consider digitalis is very seasonably employed, and even when lymph is effused. For as our object is now to effect the absorption of the lymph; and as we know the powers of the circulation and absorption are in an inverse relation, and therefore whatever depresses the former increases the latter, the depressing influence of digitalis on the circulation promotes the energy of the absorbents, which is required to remove the lymph. But when all hope of the lymph being removed is at an end, the time is now arrived for laying aside an agent whose direct effort is to bring the heart into a condition most favorable for a result the least to be desired, viz., adhesion of the opposite pericardial surfaces; for of course the less motion there is of the organ the less interruption will there be to this adhesion. So much for the impolicy of continuing the use of digitalis in pericarditis. And there are also objections to continuing its use in the early stage of valvular disease? Here, too, I would employ it, while my object is to remove the lymph by absorption. But I should expect as a consequence of its prolonged use a condition of the circulation—viz., its retardation—which would favor the deposition of lymph on the valves, which lymph would be carried away by the blood if it retained its normal form and velocity. Thus have we often seen in our *post mortem* examinations what are called vegetations on the valves of the heart, which were nothing more than deposits of fibrin, which, we had no doubt, were deposited there just when the circulation was failing, and death near at hand.

The remarks which we have just made, apply to the earlier stage of valvular disease, not to that stage when the valvular disease is established, and when nature, exerting herself to overcome an obstruction, puts forth increased efforts, which, to a certain extent, have a salutary tendency. The time has now arrived when this medicine, whose direct effect is to depress the action of the heart, is out of place, as antagonizing this salutary effort of nature. It is thus we regard it as ill-suited to that increased action of the organ which is so constant in disease of the aortic valves, as also in the regurgitant mitral orifice, which eventuates, in both cases, in eccentric hypertrophy of the left ventricle. I am convinced the results of the treatment of heart disease would be infinitely more satisfactory than they are if more care were bestowed on distinguishing the different stages of the disease, and on ascertaining and applying the suitable treatment to each stage.

To return to our treatment of rheumatism. When the acute symptoms have passed away, and all fever gone, we now conclude our treatment with bark and hydriodate of potash, or quinine; and when stiffness of joints alone remains, with warm baths. We have ever found that, as long as the disease retains any of its acute character, so long will no benefit be derived from the warm bath; but so far from it the patient generally complains that his pains had been much worse. So that, in doubtful cases, the effects of the warm bath have served me as a test of the disease, as to its having passed from the acute to the chronic stage.

In thus asserting the advantage to be derived from bleeding in acute rheumatism, we limit its advantage to what is confessedly acute rheumatism; for we have heard physicians say that bleeding had not succeeded in cases in which they had employed it; and when they described the cases we were not surprised at the failure of which they complained. The cases were such as we doubt much if they can be admitted into the category of acute rheumatism, although the two diseases have at least many local features of resemblance; but the constitutional symptoms are widely different. These cases are such as are designated diffuse inflammation, many of which we have met with as a complication of fever, and which we have described in *The Dublin Medical Journal*, Vol. XII, p. 187, in the following terms: 'We alluded to the occurrence of diffuse inflammation in some cases of this fever; we have had several instances of it, and had reason always to regard it as a most fatal complication. It exhibited itself most commonly in the form of tumefaction of the joints, sometimes with a slight erythematous blush. The knees, ankles, and wrists were the most common seat of this affection; the constitutional symptoms were in general, extreme prostration of the powers of the system, delirium, small weak pulse, diarrhœa, tympanitic abdomen, and an indescribable anxiety. On examination of the parts affected with inflammation, the tumefied joints were generally found to contain purulent matter of a thin, greenish, unhealthy character; and in some cases the cartilages were either in whole or in part destroyed, leaving the ends of the bones denuded and rough.'

We know that such cases as we have thus described have been taken or mistaken for cases of acute rheumatism, and have been treated accordingly; we can hardly wonder that success did not attend such treatment. If such cases have any title to be designated rheumatism, they should be designated typhoid or asthenic rheumatism. In speaking of venesection as an essential element of our treatment we deny the justice of identifying our treatment with that of Bouillaud who bled *coup sur coup;* while Dr. Griffin's remarks would at least seem to imply this. At a time when we exercised less reserve in ordering our patients, affected with rheumatism, to be blooded, we never carried it to anything like the length of the distinguished French physician.

We have already observed we gave a fair trial to every other mode of treating the disease, viz., the alkaline treatment, the citric acid treatment, the treatment with opium freely exhibited, the treatment with colchicum alone, and the treatment with bark, with hydriodate of potash, and none has approached the plan we have recommended, in the shortness of time required, nor has any been more certain in its results. And time and ample experience have established its pretensions with us."—*Am. Jour. of Med. Science.*

